# Analgesia and sedation strategies in neonates undergoing whole-body therapeutic hypothermia: A scoping review

**DOI:** 10.1371/journal.pone.0291170

**Published:** 2023-12-07

**Authors:** Mahima Joshi, Javed Muneer, Lawrence Mbuagbaw, Ipsita Goswami

**Affiliations:** 1 Faculty of Sciences, McMaster University, Hamilton, Ontario, Canada; 2 Department of Pediatrics, McMaster University, Hamilton, Ontario, Canada; 3 Health Research Methods, Evidence and Impact, McMaster University, Hamilton, Ontario, Canada; Van Yuzuncu Yil University, TURKEY

## Abstract

**Background:**

Therapeutic hypothermia (TH) is a widely practiced neuroprotective strategy for neonates with hypoxic-ischemic encephalopathy. Induced hypothermia is associated with shivering, cold pain, agitation, and distress.

**Objective:**

This scoping review determines the breadth of research undertaken for pain and stress management in neonates undergoing hypothermia therapy, the pharmacokinetics of analgesic and sedative medications during hypothermia and the effect of such medication on short- and long-term neurological outcomes.

**Methods:**

We searched the following online databases namely, (i) MEDLINE, (ii) Web of Science, (iii) Cochrane Library, (iv) Scopus, (v) CINAHL, and (vi) EMBASE to identify published original articles between January 2005 and December 2022. We included only English full-text articles on neonates treated with TH and reported the sedation/analgesia strategy used. We excluded articles that reported TH on transport or extracorporeal membrane oxygenation, did not report the intervention strategies for sedation/analgesia, and reported hypoxic-ischemic encephalopathy in which hypothermia was not applied.

**Results:**

The eligible publications (n = 97) included cohort studies (n = 72), non-randomized experimental studies (n = 2), pharmacokinetic studies (n = 4), dose escalation feasibility trial (n = 1), cross-sectional surveys (n = 5), and randomized control trials (n = 13). Neonatal Pain, Agitation, and Sedation Scale (NPASS) is the most frequently used pain assessment tool in this cohort. The most frequently used pharmacological agents are opioids (Morphine, Fentanyl), benzodiazepine (Midazolam) and Alpha2 agonists (Dexmedetomidine). The proportion of neonates receiving routine sedation-analgesia during TH is center-specific and varies from 40–100% worldwide. TH alters most drugs’ metabolic rate and clearance, except for Midazolam. Dexmedetomidine has additional benefits of thermal tolerance, neuroprotection, faster recovery, and less likelihood of seizures. There is a wide inter-individual variability in serum drug levels due to the impact of temperature, end-organ dysfunction, postnatal age, and body weight on drug metabolism.

**Conclusions:**

No multidimensional pain scale has been tested for reliability and construct validity in hypothermic encephalopathic neonates. There is an increasing trend towards using routine sedation/analgesia during TH worldwide. Wide variability in the type of medication used, administration (bolus versus infusion), and dose ranges used emphasizes the urgent need for standardized practice recommendations and guidelines. There is insufficient data on the long-term neurological outcomes of exposure to these medications, adjusted for underlying brain injury and severity of encephalopathy. Future studies will need to develop framework tools to enable precise control of sedation/analgesia drug exposure customized to individual patient needs.

## Introduction

Neonatal encephalopathy related to intrapartum events accounts for 6.1 million years lived with disability and 50.2 million disability-adjusted life years globally [1]. In moderate to severe encephalopathy, therapeutic hypothermia (TH) started within 6 hours of birth prevents neurodevelopmental impairment with a number needed to treat of 7 [2, 3]. A significant portion of neuronal cell death occurs during the secondary phase of brain injury, which starts approximately 4–6 hours after the primary hypoxic-ischemic insult, rendering it preventable by TH [4]. Whole-body hypothermia is a widely accepted treatment for neonatal hypoxic-ischemic brain injury [2]. The core body temperature of the affected neonate is reduced using a servo-controlled device, maintained at 33–34°C for 72 h, followed by slow rewarming to normal temperatures [5].

Human biochemical and physiologic processes are tightly regulated at specific body temperatures [6]. Hence, TH alters homeostasis by inducing peripheral vasoconstriction, bradycardia, reduced cardiac output, and metabolic rate [7]. Counter-regulatory processes activated in neonates include brown fat non-shivering thermogenesis [8] and non-exercise activity thermogenesis in the form of restlessness, agitation, crying and shivering [9]. Concordant activation of the hypothalamic-pituitary-adrenal axis and release of cortisol is the body’s innate response to achieve homeostasis [10]. Appropriate containment of such innate stress response is essential to provide compassionate care at the temperatures required for effective neuroprotection. Preventing stress is essential for limiting secondary brain injury after hypoxic brain insult [10–12]. Untreated pain during the neonatal period negatively impacts future pain sensitivity [13, 14], brain development [15], and functional outcomes [16, 17]. Nevertheless, achieving optimal sedation and pain control in this cohort is challenging due to: (i) the inability of neonates to express pain sensation verbally, (ii) multi-organ injury and hypothermia alter the pharmacokinetics of most drugs, and (iii) the potential deleterious effect of cumulative doses of sedatives and analgesics on the developing brain [18, 19]. Most centres adapt the use of medications reported in randomized controlled cooling trials [2]. This population has no standard practice guidelines for stress and pain management. A preliminary search revealed three reviews related to the topic, which included a narrative review [20] and an attempted systematic review which found no randomized controlled trials (RCT) on the topic [21]. Another recent Cochrane review concluded there is limited evidence to establish the benefits versus harm of pharmacological and non-pharmacological interventions for the management of pain and sedation in newborn infants undergoing TH for hypoxic-ischemic encephalopathy. However, these reviews were narrow in scope, so a more detailed description and evaluation of the current sedation and analgesia practices is needed using a systematic methodology.

This scoping review was undertaken to map and report the breadth of existing literature around the key concepts of sedation and analgesia during induced hypothermia in neonates. The four questions that guided this review are: (i) What pain and stress assessment tools are currently available for neonates undergoing whole-body hypothermia? (ii) What pain and stress management strategies are currently used for neonates undergoing hypothermia treatment? (iii) What is the extent of knowledge regarding alterations in the pharmacokinetics of analgesia and sedative medications during hypothermia in neonates? (iv) How do analgesia and sedation affect short- and long-term outcomes of neonates who undergo TH?

## Methods

We adapted the Arksey and O’Malley framework modified by Levac et al. [22] and the Joanna Briggs Institute [23] to conduct this scoping review. An a priori scoping review protocol was registered with the Open Science Framework on December 13th, 2022 (https://doi.org/10.17605/OSF.IO/8S2U3). Duplicates were removed by uploading all citations into EndNote version 20.3 (Clarivate Analytics, PA, USA). Two independent reviewers [JM and MH] screened titles/abstracts to retrieve potentially relevant sources. Their citation details were imported into the Covidence systematic review software (Veritas Health Innovation, Melbourne, Australia) available at www.covidence.org. Two independent reviewers [JM and MH] analysed the full text articles of the selected citations. Disagreements between the reviewers were resolved through discussion or by an additional reviewer [IG]. We followed the Preferred Reporting Items for Systematic Reviews and Meta-analyses extension for scoping review (PRISMA-SCR) checklist throughout this manuscript to present the details of the result [*S1 Appendix*] [24].

### Search strategy

Text words within the titles/abstracts of identified articles and index terms were used to develop a comprehensive search strategy for the following online database: (i) MEDLINE/PubMed, (ii) Web of Science, (iii) Cochrane Library, (iv) SCOPUS, (v) CINAHL, and (vi) EMBASE (*S2 Appendix).* A full search was undertaken on January 5th, 2023. Articles published in English between January 2005 and December 2022 were included. Inclusion criteria: (i) available in full text, (ii) focus on neonates treated with TH and (iii) reports the sedation/analgesia strategy used. Studies were excluded if they: (i) reported TH on transport or extracorporeal membrane oxygenation, (ii) did not report the intervention strategies for sedation/analgesia, (iv) reported hypoxic-ischemic encephalopathy in which hypothermia was not applied.

### Eligibility criteria

This scoping review considered randomized controlled trials, non-randomized controlled trials, pharmacokinetic studies, analytical observational cohorts, case-control, and cross-sectional surveys. We excludedreview articles,conference abstracts, case reports/series, commentaries or editorial articles, opinion papers, animal studies and preclinical studies.

### Participants

Neonates with a gestational age of > 35 weeks at birth who experienced perinatal asphyxia and received TH within the first four days of life were considered for inclusion in the review.

### Concept

"Sedation" was defined as a medically induced temporary depression of consciousness before and/or during interventional procedures that cause pain or discomfort in patients with the primary aim of relieving distress [25]. We excluded "palliative sedation therapy," defined as medication to relieve intolerable and refractory pain by reducing patient consciousness. Variations of the concept of sedation were included, such as "mild sedation," "intermittent sedation," "continuous sedation," and "deep sedation". "Analgesia" is any pharmacological agent or nonpharmacological procedure that mitigates the sensation of pain without reducing consciousness. Other variations of the concept of analgesia were included, such as "anti-nociception," "pain medications," and "pain control".

### Context

TH is a neuroprotective strategy adopted when a hypoxic-ischemic brain injury is suspected around the time of birth. Different variations of the terminology, such as "passive cooling," "active cooling," selective head cooling," "whole-body hypothermia," "targeted temperature management," and "induced hypothermia."

### Data extraction and presentation

Two independent reviewers [JM and MJ] extracted data using a data-extraction tool (*S3 Appendix)* and presented in tables and figures aligned to the review questions accompanied by a narrative summary. We grouped the studies by the review questions they provided information on and summarized the type of study design, aim statement, sample sizes, and year of publication.

## Results

### Study characteristics

**Fig 1:**
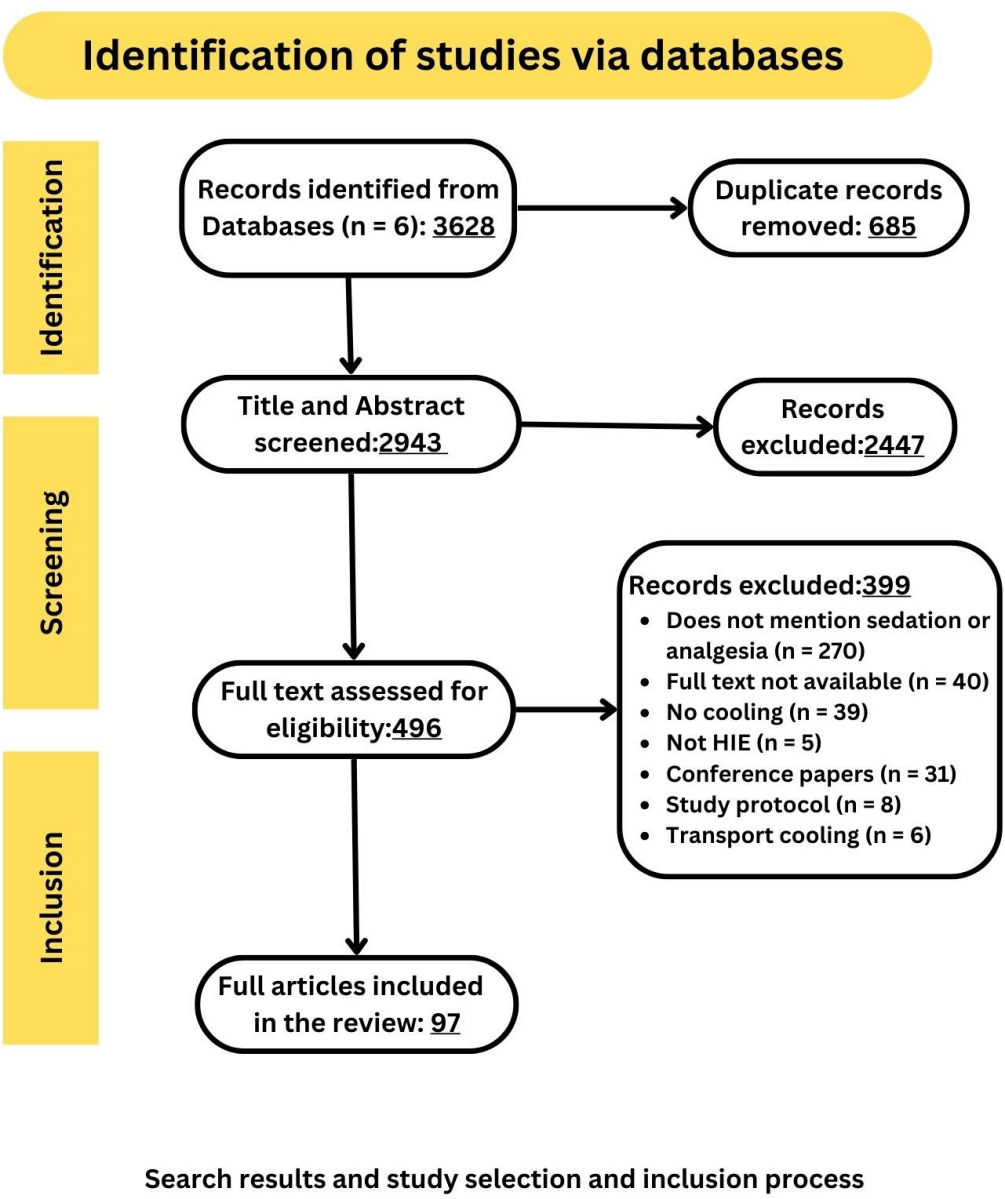
Flow diagram depicting the steps of search process, study selection and inclusion of studies that met the predefined inclusion criteria.

A total of 97 studies met the inclusion criteria (**Fig 1**). Among 97 articles, 15 articles met the criteria for review question 1, 97 articles met the criteria for review question 2, 14 articles met the criteria for review question 3,18 articles met the criteria for review question 4 (**Table 1**). Of the included articles, 72 were cohort studies; two were non-randomized experimental studies, four were pharmacokinetic studies; one was a single-arm dose escalation feasibility trial, five were cross-sectional surveys, and 13 were randomized control trials.

**Table 1:** Characteristics of the included studies.

Authors	Aim of the Study	Study Design	Sample Size	Publication Year	Review Question answered
Aker *et al*. [40]	To evaluate the neuroprotective effect of TH induced by phase-changing material on MRI biomarkers in infants with HIE in a low-resource setting.	Randomized controlled trial	N = 50	2020	Questions 1, 2
Azzopardi *et al* [90]	To evaluate whether TH improves neurodevelopmental outcome	Randomized controlled trial	N = 325	2009	Question 2
Balduini *et al*. [63]	To test the safety, pharmacokinetics, and dosage of enteral melatonin in hypothermic neonates	Pharmacokinetic Study	N = 5	2019	Question 3
Barta *et al*. [91]	To study the effects of gestational and postnatal age on metabolite levels in MR spectroscopy.	Retrospective Cohort	N = 484	2022	Question 2
Bersani *et al*. [92]	To investigate the predictive role of urinary S100B concentrations in HIE infants receiving TH	Prospective case-control study	N = 88	2022	Question 2
Berube *et al*. [48]	To describe the use of sedatives and analgesics during TH in encephalopathic neonates and association between medication exposure and hospital outcomes.	Cross-sectional Survey	N = 125	2020	Questions 2,4
Bharadwaj *et al*. [93]	To evaluate whether whole-body cooling using gel packs in infants with encephalopathy would reduce death or developmental delay at six months	Randomized controlled trial	N = 130	2012	Question 2
Bonifacio *et al*. [94]	To compare the association between perinatal events and the pattern of brain injury on early MRI in newborn infants with and without TH for HIE	Prospective cohort study	N = 60	2011	Question 2
Botondi *et al*. [95]	To investigate the potential effects of perinatal asphyxia on longitudinal presepsin urine levels.	Case-Control Study	N = 76	2022	Question 2
Brotschi *et al*. [57]	To study whether temperature management during TH correlates with the severity of brain injury in term infants with HIE	Registry database study	N = 55	2017	Question 2
Cainelli *et al*. [96]	To study the long-term cognitive outcomes of infants with HIE who received TH	Prospective case-control study	N = 40	2021	Question 2
Cilla *et al*. [97]	To describe normal C-reactive protein levels of newborns diagnosed with HIE	Prospective cohort study	N = 225	2020	Question 2
Cornet *et al*. [98]	To assess the prognostic significance of an early conventional EEG on seizure risk in neonates undergoing therapeutic hypothermia.	Retrospective Cohort Study	N = 323	2018	Question 2
Cosnahan *et al*. [29]	To evaluate the impact of changing TH sedation protocol from intermittent morphine to dexmedetomidine on efficacy and safety parameters.	Retrospective Cohort Study	N = 70	2021	Questions 1,2,4
Craig *et al*. [42]	To assess nurse attitudes to the provision of TH concerning perceptions about baby pain/sedation, the need for nurse and parent education, decision-making about the initiation of TH, and barriers.	Cross-sectional Survey	N = 219	2017	Question 2
Cseko *et al*. [73]	To assess the predictive value of EEG in hypothermia-treated HIE infants.	Retrospective Cohort Study	N = 70	2013	Questions 2,4
Dassios *et al*. [99]	To describe how TH could impact respiratory function in mechanically ventilated newborn infants.	Retrospective Cohort Study	N = 31	2014	Question 2
Debillon *et al*. [100]	To test the practicability and safety of whole-body cooling in term neonates with moderate-to-severe HIE and to report outcomes.	Prospective cohort study	N = 25	2003	Questions 2, 4
Dingley *et al*. [39]	To test the feasibility of xenon gas as a neuroprotective therapy in cooled infants.	Single Arm dose Escalation study	N = 14	2014	Questions 1, 2
Dingley *et al*. [101]	To describe the impact of a transportable closed-circuit Xenon delivery system on the ability to deliver Xe within 5 hours.	Randomized controlled trial	N = 32	2015	Question 2
Eicher *et al*. [102]	To compare the efficacy of TH to normothermia for 48 hours in neonates initiated within 6 hours of a hypoxic-ischemic event.	Randomized controlled trial	N = 55	2005	Question 2
Elliott *et al*. [71]	To describe local experience with dexmedetomidine and fentanyl in neonates undergoing TH for HIE.	Retrospective Cohort Study	N = 166	2022	Questions 2,4
Favie *et al*. [65]	To describe the pharmacokinetics of morphine and its metabolites in encephalopathic neonates treated with TH and develop dosing guidelines.	Pharmacokinetic Study	N = 244	2019	Question 3
Favie *et al*. [64]	To better understand underlying pharmacokinetic principles to guide drug dosing.	Pharmacokinetic Study	N = 192	2020	Question 3
Filippi *et al*. [103]	To evaluate whether the efficacy of moderate hypothermia can be increased by concomitant topiramate treatment.	Randomized controlled trial	N = 64	2012	Question 2
Forman *et al*. [104]	To assess the feasibility and reliability of non-invasive cardiac output monitoring and near-infrared spectroscopy.	Prospective cohort study	N = 20	2017	Question 2
Fredriksson *et al*. [35]	To evaluate pain assessment routines, assess pain treatment strategies in newborn infants undergoing TH, and provide pharmacokinetic explanations of possible mechanisms that can affect pain treatment.	Retrospective Cohort Study	N = 17	2013	Questions 1,2,3
Frymoyer *et al*. [66]	To describe the pharmacokinetics of morphine in neonates with HIE receiving hypothermia.	Pharmacokinetic Study	N = 20	2017	Question 2, 3
Gagne-Loranger *et al*. [59]	To describe severity of brain injury and mortality in newborns receiving TH about the degree of encephalopathy on admission.	Prospective cohort study	N = 215	2015	Question 2
Garcia-Alix *et al*. [105]	To investigate the circumstances surrounding end-of-life decisions of infants with HIE and examine changes over ten years.	Retrospective Cohort Study	N = 70	2013	Question 2
Garvey *et al*. [106]	To describe early cerebral oxygenation values and their evolution over the first days of life in infants with HIE and to determine early measures to predict short-term outcomes.	Prospective cohort study	N = 58	2022	Question 2
Garvey *et al*. [107]	To describe early, continuous, noninvasive measures of cardiac output and evolution over time in infants with HIE.	Prospective cohort study	N = 44	2022	Question 2
Gauda *et al*. [67]	To determine a safe dose of clonidine for infants with HIE undergoing TH.	Pharmacokinetic Study	N = 40	2022	Question 3
Goulding *et al*. [108]	To describe heart rate variability in neonatal HIE and correlate HRV with EEG grade of HIE and neurodevelopmental outcome.	Retrospective Cohort Study	N = 61	2015	Question 2
Guidotti *et al*. [109]	To evaluate the antiepileptic effect of hypothermia and its association with neurological outcomes in infants with moderate and severe HIE.	Retrospective Cohort Study	N = 72	2016	Question 2
Gundersen *et al*. [38]	To examine the effect of opioid administration during TH on the neurodevelopmental outcome and time to extubation after sedation ended.	Prospective cohort study	N = 258	2021	Questions 1, 2, 4
Hagmann *et al*. [52]	To evaluate the practice of TH amongst Swiss neonatologists and pediatric intensive care specialists.	Cross-sectional Survey	N = 18	2011	Question 2
Oliveira *et al*. [51]	To describe the feasibility of TH using a low-cost servo-controlled cooling device and the short-term outcomes of the cooled babies.	Prospective cohort study	N = 82	2018	Question 2
Horn *et al*. [110]	Describe the use, efficacy, and physiologic impact of an inexpensive servo-controlled cooling fan blowing room-temperature air.	Prospective cohort study	N = 10	2009	Question 2
Horn *et al*. [111]	To determine whether clinical assessment at age 3-5h predicts a severely abnormal aEEG at 48h or death in cooled infants.	Prospective cohort study	N = 41	2013	Question 2
Horn *et al*. [112]	To determine if early clinical examination could predict either an abnormal aEEG at age 6 hours or moderate-severe HIE presenting within 72 hours of birth.	Prospective cohort study	N = 60	2013	Question 2
Horn *et al*. [113]	To describe and evaluate a simple method of neuroprotective hypothermia for infants with HIE.	Prospective cohort study	N = 5	2010	Question 2
Howlett *et al*. [114]	To describe the relationship between autoregulation and neurologic injury in HIE.	Prospective cohort study	N = 44	2013	Question 2
Jain *et al*. [115]	To evaluate and compare early EEG power and EEG as predictors of MRI injury in neonatal HIE.	Retrospective Cohort Study	N = 78	2017	Question 2
Kali *et al*. [68]	To assess whether the benefits of TH could be improved upon by adding morphine to TH	Randomized controlled trial	N = 45	2021	Questions 2, 3, 4
Lago *et al*. [34]	To analyze data on current analgesia and sedation practices during TH in Italian NICU	Cross-sectional Survey	N = 70	2020	Questions 1, 2
Lakatos *et al*. [116]	To determine whether the presence of intracranial hemorrhage on MRI alongside the signs of HIE has an impact on prognosis	Retrospective Cohort Study	N = 108	2019	Question 2
Lin *et al*. [117]	To determine the efficacy of mild hypothermia via selective head cooling as a neuroprotective therapy in term infants with perinatal asphyxia.	Randomized controlled trial	N = 58	2006	Question 2
Liow *et al*. [118]	To examine the association of pre-emptive morphine infusion during TH on brain injury and neurodevelopmental outcomes	Prospective cohort study	N = 169	2020	Question 4
Liu *et al*. [58]	To test cerebral autoregulation in newborns with HIE.	Prospective cohort study	N = 79	2021	Question 2
Lori *et al*. [119]	To explore whether continuous somatosensory evoked potentials and video EEG accurately predict brain injury in neonates with HIE	Prospective cohort study	N = 31	2022	Question 2
Lucke *et al*. [120]	To assess MR spectroscopy in neonates with HIE within 18–24 h of initiating TH and at 5–6 days post TH.	Prospective cohort study	N = 11	2019	Question 2
Lugli *et al*. [33]	To assess the safety of fentanyl during TH by evaluating adverse effects and possible correlation with the neurodevelopmental outcome.	Prospective cohort study	N = 45	2022	Question 1,2,3,4
Mahdi *et al*. [72]	To assess the association between the level of exposure to opioids and temperature, with EEG background activity post-TH and MRI brain injury in neonates with HIE.	Retrospective Cohort Study	N = 31	2022	Questions 2,4
Mann *et al*. [121]	To compare the ion gap with base excess and lactate for predicting neurologic outcome measured by MRI in newborns with HIE.	Retrospective Cohort Study	N = 39	2012	Question 2
Markus *et al*. [122]	To analyze enteral and parenteral nutritional supply during and after TH	Retrospective Cohort Study	N = 135	2021	Question 2
Massaro *et al*. [123]	To evaluate whether impaired cerebral autoregulation during TH and rewarming relates to outcomes in HIE newborns.	Prospective cohort study	N = 36	2015	Question 2
McAdams *et al*. [30]	To study the pharmacokinetics and safety of dexmedetomidine	Pharmacokinetic Study	N = 7	2020	Questions 1,2, 3
McDonough *et al*. [124]	To predict epilepsy in neonates after selective head cooling.	Retrospective Cohort Study	N = 50	2017	Question 2,4
Meder *et al*. [74]	To investigate the predictive accuracy of aEEG background activity to predict long-term neurodevelopmental outcomes in neonates with HIE receiving TH	Retrospective Cohort Study	N = 206	2022	Questions 2,4
Montaldo *et al*. [125]	To assess the electrocardiography changes during TH and rewarming period in encephalopathic infants with long-term adverse neurological outcomes.	Prospective cohort study	N = 64	2018	Question 2
Natarajan *et al*. [53]	To correlate the early EEG background pattern with clinical course and outcome in mild HIE who underwent TH	Retrospective Cohort Study	N = 29	2022	Questions 2,4
Natarajan *et al*. [60]	To study the association between opioid exposure during TH for HIE and in-hospital outcomes.	Retrospective Cohort Study	N = 1484	2022	Questions 2,4
Natarajan *et al*.[62]	To evaluate the association between sedation-analgesia during the initial 72 h and death/disability at 18 months of age in HIE.	Secondary Analysis of clinical trial	N = 208	2018	Question 4
Naveed *et al*. [31]	To evaluate the safety and efficacy of dexmedetomidine compared with fentanyl in neonates with HIE undergoing TH	Retrospective Cohort Study	N = 45	2022	Questions 1, 2, 3, 4
Niezen *et al*. [126]	To assess the predictive value of aEEG and near-infrared spectroscopy during TH	Retrospective Cohort Study	N = 39	2018	Question 2
Nitzan *et al*. [45]	To study changes in oxygenation in neonates after rewarming following moderate therapeutic hypothermia for neonatal encephalopathy.	Retrospective Cohort Study	N = 28	2019	Questions 2 & 4
O’Mara *et al*. [26]	To evaluate dexmedetomidine infusion’s efficacy and short-term safety for sedation in term neonates undergoing TH for HIE.	Retrospective Cohort Study	N = 19	2018	Questions 1,4
Odd *et al*. [32]	To determine whether parents cuddling infants during TH would affect cooling therapy, cardiorespiratory or neurophysiological measures.	Prospective cohort study	N = 27	2021	Questions 1, 2
Oliveira *et al*. [51]	To report current cooling practices for babies with mild encephalopathy in the UK.	Cross-sectional Survey	N = 68	2018	Question 2
Orbach *et al*. [127]	To examine the relationship between TH and seizure in neonates with HIE	Retrospective Cohort Study	N = 224	2014	Question 4
Pazandak *et al*. [128]	To describe mean arterial blood pressure, responsiveness to dopamine, and relationship to brain injury in infants with HIE undergoing TH.	Prospective cohort study	N = 18	2020	Question 2
Prempunpong *et al*. [46]	To investigate the effect of the TH on fluid balance and incidence of hyponatremia.	Retrospective Cohort Study	N = 67	2013	Question 2
Radicioni *et al*. [129]	To assess the incidence of sinovenous thrombosis in a population of asphyxiated cooled infants	Prospective cohort study	N = 37	2017	Question 2
Roka *et al*. [54]	To compare serum morphine concentrations in neonates with HIE undergoing hypothermia and normothermic infants.	Pharmacokinetic Study	N = 16	2008	Question 4
Roychoudhury *et al*. [47]	To evaluate the impact of a dedicated neonatal neurocritical care service on short-term outcomes in infants with HIE	Retrospective Cohort Study	N = 216	2019	Question 2
Saito *et al*. [130]	To investigate the time difference between peak levels of serum CRP and other inflammatory responses during TH	Prospective cohort study	N = 22	2016	Question 2
Sakhuja *et al*. [131]	To assess gastrointestinal blood flow and left ventricle output in infants with HIE during whole body TH and after rewarming.	Prospective cohort study	N = 20	2019	Question 2
Sehgal *et al*. [41]	To compare cardiac indices between asphyxiated infants and healthy controls and the correlations between strain and cardiac troponin	Retrospective Cohort Study	N = 244	2013	Question 2
Shankaran *et al*. [4]	To examine the predictive ability of stage of HIE for death or moderate/severe disability at 18 months among neonates undergoing hypothermia.	Randomized controlled trial	N = 204	2012	Question 2
Sheppard *et al*. [27]	To determine the degree to which whole-body hypothermia impacts hemodynamic and respiratory status in neonates with HIE	Retrospective Cohort Study	N = 65	2021	Questions 1,2
Simbruner *et al*. [49]	To determine the efficacy of systemic TH in term neonates with HIE compared to normothermia, to determine whether the protective effect of TH was related to the severity of HIE, and to evaluate the safety of hypothermia.	Randomized controlled trial	N = 129	2010	Question 2
Smit *et al*. [55]	To define the incidence of hearing impairment and identify factors associated with permanent hearing impairment in infants subjected to TH for moderate or severe neonatal encephalopathy.	Prospective cohort study	N = 108	2013	Question 2
Steiner *et al*. [132]	To determine the predictive power of the combined use of neurophysiological, near-infrared spectroscopy and MRI for long-term outcome prediction in neonates with HIE	Prospective cohort study	N = 56	2022	Question 2
Suppiej *et al*. [133]	To study associations between neonatal routine parameters recorded in NICU and the development of severe outcomes.	Prospective cohort study	N = 83	2021	Question 2
Surkov *et al*. [70]	To compare cerebral blood flow indexes and results of treatment for HIE between groups of full-term infants who received dexmedetomidine versus other sedatives during TH	Prospective case-control study	N = 205	2019	Questions 2,3,4
Surmeli Onay *et al*. [28]	To evaluate the effect of aminophylline on estimated glomerular filtration rate, urine output, and incidence and severity of AKI in newborns with HIE under TH	Case-Control Study	n = 34	2021	Questions 1,3
Tanaka *et al*. [56]	To describe a novel aEEG pattern in infants with HIE and to assess the clinical significance.	Retrospective Cohort Study	N = 46	2020	Question 2
Thoresen *et al*. [10]	To document cardiovascular changes associated with TH and rewarming in such infants.	Prospective cohort study	N = 9	2000	Question 2
Tran *et al*. [134]	To evaluate whether phase-changing material can be used for TH in low-resource settings.	Prospective cohort study	N = 52	2021	Question 2
Uner *et al*. [135]	To translate the N-PASS scoring system and assess the use of this scoring system on neonates undergoing TH	Prospective cohort study	N = 17	2019	Question 1
Van den Broek *et al*. [36]	To evaluate midazolam’s anticonvulsant effectiveness and hemodynamic safety in hypothermic newborns and to provide dosing guidance.	Pharmacokinetic Study	N = 53	2015	Questions 1,2, 3,4
Vega-Del-Val *et al*. [136]	To examine adherence to management standards during TH of infants with HIE	Retrospective Cohort Study	N = 133	2022	Question 2
Vergales *et al*. [137]	To study the association between heart rate variability and adverse short-term outcomes in neonates with HIE	Prospective cohort study	N = 37	2014	Question 2
Welzing *et al*. [37]	To investigate the disposition of midazolam in asphyxiated neonates with TH	Pharmacokinetic Study	N = 9	2013	Questions 1,2, 3
Wisnowski *et al*. [61]	To characterize the effects of hypothermia on MR spectroscopy	Prospective cohort study	N = 40	2016	Question 2
Youn *et al*. [138]	To analyze whether earlier hypothermia improves hospital outcomes in survivors who underwent TH when compared with late TH	Case-Control Study	N = 40	2016	Question 2

Abbreviations: TH, Therapeutic Hypothermia, HIE, Hypoxic Ischemic Encephalopathy, EEG, Electro-encephalogram, aEEG, amplitude Electro-encephalogram

### Question 1. What pain and stress assessment tools are currently available?

Only 15 full-text articles reported using standardized pain scales in neonates undergoing TH. The most commonly used tool was the Neonatal Pain, Agitation, and Sedation Scale (NPASS) in seven studies [26–32]. Other tools included the EDIN scale (Échelle de Douleur et discomfort du Nouveau-né) [3 studies] [33–35], COMFORT scale [2 studies] [34, 36], Visual Analog scale (VAS) scores [1 study] [36], Hartwig score [1 study] [37], Facial Pain rating scale [3 studies] [34, 38, 39], and Neonatal Infant Pain Scale [1 study] [40]. Lago *et al*. reported that 78% of the centers surveyed used a standardized pain scale to monitor neonates undergoing TH [34]. McAdams *et al*. reported the use of the Bedside Shivering Assessment Tool [30].

### Question 2. What type of pain and stress management strategies are currently being used?

**Fig 2:**
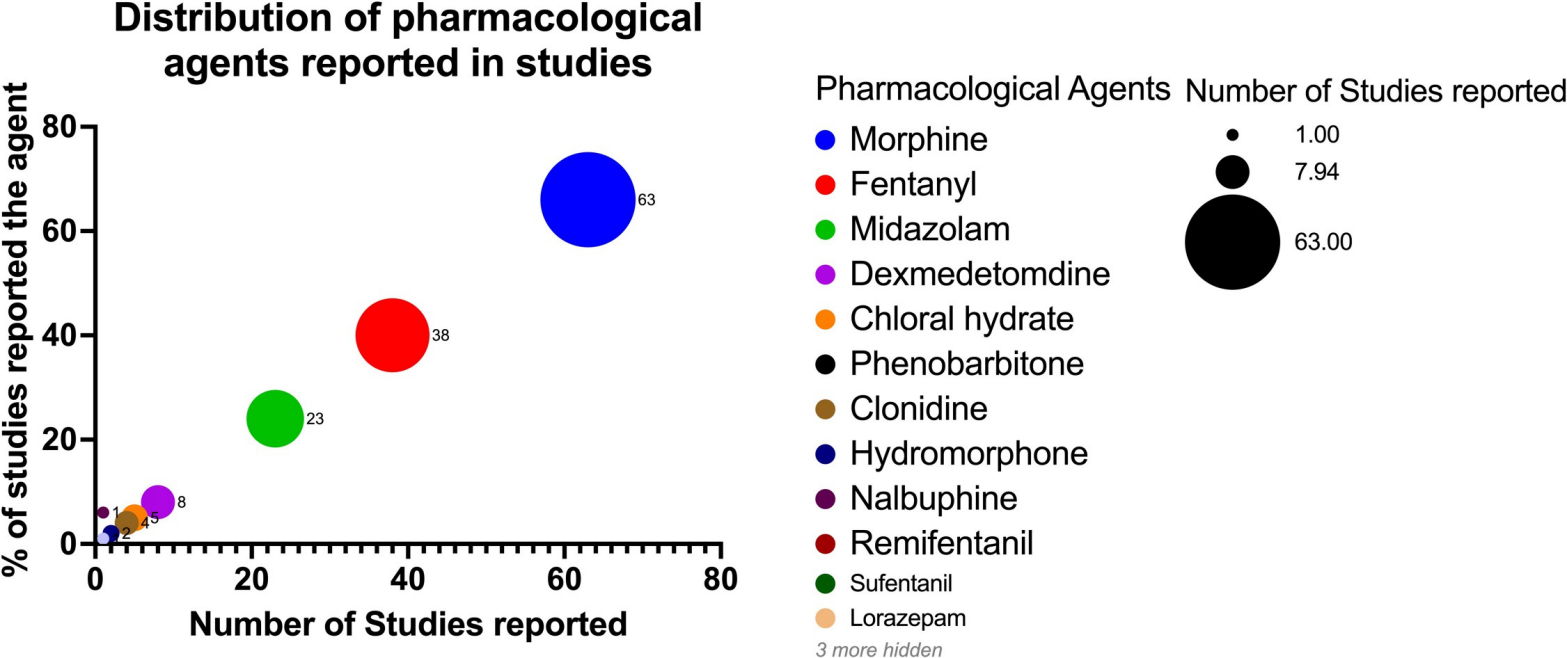
Enumerates the list of pharmacological agents that have been used in studies for the purpose of sedation and analgesia management during therapeutic hypothermia and the frequency with which individual agents have been used in different studies.

Ninety-four articles reported using medications for comfort, and one small study reported no use of routine sedation during TH [41]. In a qualitative survey, the perception of "discomfort" during TH was more prevalent among NICU nurses from centres that did not use medications (62%) compared to centres using routine morphine during TH (20%) [42]. Few studies have explored potential nonpharmacological comfort measures, such as a feasibility trial of 30-minute maternal holding during TH in non-ventilated infants [43]. The CoolCuddle Study reported physiological and temperature stability with no change in pain scales or analgesic needs during a 2-hour-cuddle for neonates undergoing TH [32]. The majority of studies reported the use of medications for sedation/analgesia during TH. Frequently used agents are Morphine, Fentanyl, Midazolam, and Dexmedetomidine (**Fig 2**). Five studies report the use of sedation but do not mention the agent used [32, 40, 44–46], while two studies use generic names such as "opiates" and "benzodiazepines" instead of identifying the specific medication [47, 48]. **Table 2** lists the different dose ranges of medications used.

**Table 2:** Summary of all studies which provide the dosage of individual medication used for sedation analgesia during therapeutic hypothermia in neonates.

Study	Opiates	Benzodiazepines	Alpha blockers	Others	Dosage
Lin *et al [117]*				PB	20 mg/kg bolus and then 5 mg/kg per day for a minimum of 72 h
Welzing *et al [37]*	Fe	Mi			Continuous infusion of Mi at a dose of 30–100 μg/kg/h. Fe infusion 1–4μg/kg/h.
Debillon *et al [100]*	Fe,Mo,SuNa				Fe median dose of 1.5μg/kg/h, Mo median dose of 1.6 mg/kg/day, suFe dose 0.5μg/kg/h, nalbuphine dose 50μg/kg/h.
Montaldo *et al [125]*	Fe				Infusion 1–3μg/kg/h.
Frymoyer *et al [66]*	Mo				Infusion started at 20μg/kg/h, and reduced to 10μg/kg/h. at 24h of hypothermia treatment. Or Starting Mo dose of 40μg/kg q6h over 10mins. Additional intermitted boluses were 50–100μg/kg.
Niezen *et al [126]*	Mo	Mi			Routine sedation with Mo 10 μg/kg/h. Mi loading 0.05 mg/kg bolus, maintenance 0.1mg/kg/h if sedation
Lucke *et al [120]*	Mo				Infusion 10–20 μg/kg/h
McDonough *et al [124]*		Mi			Infusion 0.01 mg/kg/h
Simbruner *et al [49]*	Fe,Mo				0.1mg/kg Mo q4h or an equivalent dose as continuous infusion. Fe in an equivalent dose.
McAdams *et al [30]*			Dex		Infusion 0.2 μg/kg/h increased to a max of 0.4 μg/kg/h in 2.5h
Cainelli *et al [96]*	Fe				Infusion 1μg/kg/h with bolus as needed
Lori *et al [119]*	Fe				Infusion 1–2μg/kg/h with bolus as needed
Suppiej *et al [133]*	Fe				Infusion 1–2μg/kg/h with bolus as needed
Dassios *et al [99]*	Mo				Infusion 10–20μg/kg/h
Tran *et al* [134]	Mo				Infusion 10μg/kg/h
Filippi *et al [103]*	Fe				Infusion 1μg/kg/h
Dingley *et al [29]*	Mo				Infusion 20μg/kg/h
Dingley *et al [101]*	Mo				Infusion 20μg/kg/h
Kali *et al [68]*	Mo		Cl	PB	Mo Infusion 25μg/kg/h
Horn *et al*. [110]	Mo				Infusion 8μg/kg/h
Horn *et al*. [111]	Mo		Cl		Boluses: 2.5 μg/kg of clonidine (0.5 mL/kg of prepared suspension) was given via nasogastric tube q8 h intervals, and if the shivering persisted, then 0.1 mg/kg of intravenous Mo
Vanden Broek *et al* [36]		Mi			infusion of 0.05–0.1 mg/kg/h without a loading dose.
Tanaka *et al [56]*	Fe	Mi			infusion of Fe (1–3 μg/kg/h) and Mi (0.1–0.3 mg/kg/h)
Onay *et al [28]*	Fe	Mi			infusion of Fe (1–5 μg/kg/h) with Mi (10–60 μg/kg/h) added if necessary
Csekë *et al*	Mo				10 μg/kg/h was started following the loading dose 0.1 mg/kg
Lakatos *et al [116]*	Mo				10 μg/kg/h was started following the loading dose 0.1 mg/kg
Surkov *et al [70]*	Mo		Dex	Di SO	dexmedetomidine infusion 0.5 μg/kg/h
					Mo loading dose of 50 μg/kg followed by maintaining dose of 10–40 μg/kg/h, in monotherapy or in combination with SO in dose of 50–100 mg/kg or/and Di in dose of 0.05–0.1 mg/kg every 4–6 hours if needed
Naveed *et al [31]*	Fe		Dex		Fe infusions was 0.84± 0.24μg/kg/h with a maximum dose of 1.47± 0.74 μg/kg/h. The mean initial dose of Dex infusion was 0.16± 0.06μg/kg/h with a maximum dose of 0.27± 0.12 μg/kg/h.
Garcia-Ali *et al [105]*	Fe	Mi			Fe 3–5 μg/kg/h, Mi 0.05-2mg/kg/h
Lago *et al [34]*	Fe	Mi			Fe bolus 1.39 ± 0.86μg/kg; intervals between boluses 4.43 ± 1.75 h,Mo bolus 70.0 ± 30.0μg/kg; intervals between boluses 4.83 ± 1.36 h,continuous infusion 30.0 ± 20.0 μg/kg/h,Mi bolus 0.08 ± 0.07 mg/kg; intervals between boluses 3.64 ± 1.62 h continuous infusion 0.04 ± 0.02 mg/kg/h
Botondi *et al [95]*	Fe	Mi			Fe 0.5–2.5 μg/kg/h, and Mi 50–400 μg/kg/h
Bersani *et al [92]*	Fe	Mi			Fe 0.5μg/kg/h, and Mi 50μg/kg/h
Elliott *et al [71]*	Fe		Dex		Fe starting dose of 0.5 μg/kg/h with titration in 0.5 μg/kg/h increments to a maximum of 2 μg/kg/h. Dex as a first-line agent, at a starting dose of 0.2 C, with titration in 0.1 μg/kg/h increments with a typical maximum of 1 μg/kg/h. Boluses of Fe (1 μg/kg every 3 h) are given as needed for significant agitation, and sedation infusions are titrated to optimize comfort.
Balduini *et al [63]*	Me				First dose within 24hours of life with a target of 12hours of life or less. The drug was infused via enteral route through an OG tube at a dose of 0.5mg/kg. The infusion started 1hour after the neonates reached the target temperature
Meder *et al [74]*	Mo	Mi			First-line sedation was Mo infusion (loading dose of 100 μg/kg, followed by continuous infusion of 10 μg/kg/h) adjusted based on response. Second-line was Mi (boluses of 100 μg/kg/h or continuous infusion of 100 μg/kg/h as required)
O’Mara *et al [26]*	Fe		Dex		Fe started at 0.5 μg/kg/h increased to 1 μg/kg/h. Dex started at 0.3 μg/kg/h then titrated up by 0.1–0.2 μg/kg/h as needed
Mahdi *et al [72]*	Fe, Mo				intermittent Mo doses varied between 0.01 and 0.1 mg/kg while Mo infusion rates varied from 0.01 to 0.04 mg/kg/h. Intermittent doses of Fe ranged between 0.4 and 1.05 μg/kg, while perfusion rates for Fe were maintained at 1 μg/kg/h.
Cosnahan *et al [29]*	Mo		Dex		intermittent Mo doses (0.05–0.1 mg/kg) q4 h. Dexmedetomidine was initiated at 0.3 μg/kg/h and increased by 0.1 μg/kg/h increments maximum dose 2 μg/kg/h.
Horn *et al*. [110]	Mo				Infusion 8 μg/kg/h
Lugli *et al [33]*	Fe	Mi		Ket	Loading 2 μg/kg Fe bolus, followed by 0.5–2 μg/kg/h infusion rate
Favie *et al [64]*	Mo				Loading dose 50–100 μg/kg, continuous infusion 5–25 μg/kg/h
Garvey *et al [106]*	Mo				Infusion 10–20 μg/kg/h
Uner *et al [135]*	Mo Fe	Mi			Loading dose Mo 100 μg/kg/h for 2h to achieve adequate plasma level (125ng/ml). After 2h, infusion reduced to 20 μg/kg/h and then adjusted according to pain scales. If infant needed >50 μg/kg/h Mo infusion, Mi was added at a dose of 20–50 μg/kg/h. Prior to procedures, Fe boluses 1–4 μg/kg was used.
Massaro *et al [123]*	Mo				Mo 0.05 mg/kg q 4h is titrated higher
Mann *et al [121]*	Mo				Infusion 10–20 μg/kg/h
Smit *et al [55]*	Mo				Infusion 10–60 μg/kg/h
Garvey *et al [107]*	Mo				Infusion 10–20 μg/kg/h
Cornet *et al [98]*	Mo				Infusion 10–25 μg/kg/h and 5–10 μg/kg boluses
Pazandak *et al [128]*	Mo Fe		Dex		Mo initial bolus 50 μg/kg followed by a continuous infusion of 10 μg/kg/h for 12h and then decreased to 5 μg/kg/h. Continuous infusion of either Fe 0.5 μg/kg/h or Dex 0.2 μg/kg/h.
Roka *et al [54]*	Mo				Bolus dose 50–150 μg/kg followed by 5–30 μg/kg/h.
Gundersen *et al [*38]	Mo				Loading dose of 50 μg/kg followed by continuous infusion of 20 μg/kg/h.
Steiner *et al [132]*	Mo Fe	Mi			Mo 9.38 (6.59–12.21) μg/kg/h,Mi 0.08 (0.05–0.14) μg/kg/h,Fe 6.28 (4.54–9.08) μg/kg/h
Gauda *et al [67]*	Mo		Cl		Mo 0.05 mg/kg bolus,Clonidine 1 μg/kg q6h, weaned over 48h by 0.5 μg/kg/day
Markus et al [122]	Mo				Started infusion at a mean dose of 0.07 mg/kg/h. During cooling, was increased to 0.09 mg/kg/h and decreased after the end of cooling by 0.03 mg/kg/h.

Abbreviations: Fe, Fe, Mo, Mo, Mi, Mi, Cl, Clonidine, Dex, Dexmedetomidine, Su, SuFe, Na, Nalbuphine, Di, Diazepam, SO, Sodium Oxybutrias, Ke, Ketamine, PB, Phenobarbitone, Me,Melatonin

The proportion of neonates who "routinely" received medications for sedation/analgesia and shivering control varied among studies. **Table 3** summarizes all multicentre studies and surveys that describe the types of sedation/analgesia practices [34, 48–53]. Seven studies mentioned using "routine" sedation for all neonates undergoing TH [29, 37, 54–58]. Gagne-Loranger *et al*. reported the results from a prospective study (2008–2012) in which neonates were not regularly sedated during TH but received boluses of morphine only if they were uncomfortable [59]. The proportion of hypothermic neonates who received sedation in individual cohort studies varied from 44–81% [60–62]. A prospectively collected population database revealed that the median cumulative opioid dose administered during the first week of life increased by 216 μg/kg/year from 2007 to 2017, while the median duration of administration [86h] remained unchanged. The use of Fentanyl and Remifentanil has increased from 2014 to 2017 from minimal use to 40% of infants receiving additional sedatives [38].

**Table 3:** Multicentre studies that describe standard practices of units worldwide in the last decade since therapeutic hypothermia became the standard of care in neonates.

Country	No of Centres	Sample Size	Citation	Year	Findings
Italy	70	Practice Survey	*Lago et al. [34]*	2018	1st drug of choice was fentanyl (85.7%), followed by midazolam alone or combined with an opioid (28.6%). Most NICUs (71.4%) preferred using a single drug: fentanyl (58.6%), midazolam (4.3%), and morphine (7.1%). Others (28.6%) combined two drugs, usually opioids plus benzodiazepines (70.0%) or paracetamol (15.0%). The mode of administration of drugs was 38.6% only continuous infusion, 6% only bolus, and both infusion and bolus in 55%
Spain	5	Cohort (n = 133)	*Vega-del-Val et al. [136]*	2010–2019	96% of all cooled infants received sedation. Infusion (47%) and a combination of bolus and infusion (44%) were the most common. The use of morphine alone (1%), fentanyl alone (41%), morphine and fentanyl (2%), morphine or fentanyl with midazolam (53%).
United States	125	Cohort (n = 2621)	*Berube et al. [48]*	2007–2015	Opioid and benzodiazepine use during TH increased from 38% and 40% in 2008 to 68% and 53% in 2015, respectively. During the first three days, 64% of neonates received ≥1 opioid, 49% received ≥1 benzodiazepine, and 40% received both an opioid and benzodiazepine. One-third received either morphine or fentanyl, and 5% received both morphine and fentanyl. Lorazepam was administered to 17% of the neonates, and midazolam was administered to 35%. Neonates who received opioids were more likely to receive benzodiazepines than those who did not receive opioids (63% versus 24%, p ≤ 0.001). Between different centres, the prevalence of opioid and benzodiazepine exposure during postnatal days 0–3 ranged from 5 to 100% and 7 to 100%, respectively
	23	Cohort (n = 1484)	*Natarajan et al [53]*	2010–2016	The proportion of neonates who were exposed to opiates during therapy varied between 46–100% between centres. 16.2% received no opioids, 38.7% received opioids for 1–2 days and 45.2% received them for 3–5 days
United Kingdom	68	Practice Survey	*Oliveira et al. [51]*	2018	Out of 68 centres which offered TH for mild HIE, 13 (36%) sedated all cooled babies, and 20 (56%) sedated cooled babies sometimes.
Switzerland	18	Practice Survey	*Hagmann et al. [52]*	2011	73% of centres routinely provided analgesia to neonates undergoing hypothermia, with morphine being the 1st line drug. Only 9% of centres provided routine sedation, and 73% of centres provided sedation if required, with the 1st drug being midazolam in the majority of centres
Germany	24	Cohort (n = 129)	*Simbruner et al. [49]*	2001–2006	In Hypothermia efficacy trials, morphine or fentanyl was administered in 97% of the hypothermia infants and 95% of normothermic infants

Few comparative studies have assessed the best medications for sedation during TH. Neonates who were sedated with Dexmedetomidine had a decreased need for sedative boluses and shorter time to discontinuation of sedation after rewarming (1 versus 5 days; p = 0.001), shorter time to extubation (3 versus 11 days; p = 0.004), and earlier time to the resumption of feeds (9 versus 13 days; p = 0.03) than neonates who were sedated with Fentanyl [31]. Neonates who received dexmedetomidine infusion needed a higher number of breakthrough morphine (0.13 mg/kg vs 0.04 mg/kg, p = 0.001) doses but fewer cumulative morphine exposure (0.13 mg/kg vs 1.79 mg/kg, p<0.0001) compared to neonates who only received intermittent morphine for pain control during TH [29].

### Question 3. What is the extent of knowledge regarding alterations in the pharmacokinetics of analgesia and sedative medications during hypothermia in neonates?

Nine studies reported the pharmacokinetic profiles of common medications used for sedation and analgesia in neonates during TH [30, 36, 37, 63–68]. Midazolam was studied in three studies, morphine in four studies, alpha2-agonist in two studies, and melatonin in one study (**Table 4**). **Table 5** summarizes the findings from cohort studies that reported cumulative medication doses.

**Table 4:** Summary of all pharmacokinetic studies on sedative and analgesic agents used during therapeutic hypothermia in neonates.

Study	Medication	Sample Size	Results
Welzing *et al. [37]*	Midazolam	54 blood samples	• With an average infusion duration of 79 hours and dose range of 1–8 μg/kg/h, the median serum concentration was 369.3 ng/ml (36.6–3218.6 ng/ml) • 4/9 patients had elevated serum concentration (max 3218.6 ng/ml) • Median half-life during hypothermia: 9h (3–50h) • Median CL: 1.77 ml/kg/min (0.33–10.34 ml/ kg/min) • Median 1-OH-MDZ/MDZ and 4-OH-MDZ/MDZ ratios were 0.2 and 0.02 • The ratios of the hydroxy metabolites to midazolam concentration were inversely related to the half-life of midazolam
Gauda *et al. [67]*	Clonidine	29 blood samples	• median plasma clonidine level was 0.55 ng/mL (0.14–2.63 ng/mL) at a median duration of 8 h after the previous dose • q6h, the median clonidine level was 0.63 ng/mL (range 0.38–1.16) • clonidine Q8h, the median clonidine level was 0.44 ng/mL (range 0.142–2.63) • median Vd and CL of IV clonidine (1mcg/kg/q6–8h) in infants during TH was 280 L (131–475 L) and 12 L/h (95% CI 8.57–18 L) • Rebound hypertension after weaning clonidine with q4hrly doses • In comparison to the historical control group, infants treated with clonidine Q8h received 89% less PRN morphine • 92.4% of the time during TH, the CBT was within the target range (33–34°C) and a change of only 1.3°C during the 72 h of hypothermia.
Van den Broek *et al. [36]*	Midazolam	166 blood samples	• Vd, l/3.5kg: 7 (7.7), Exponent on Vd 1.02(34) • CL, l/h/3.5Kg: 0.94(23), Exponent on CL 1.65 (30) • Between subject variability V% 35%, CL% is 53% • Inotropes were administered to 89% of newborns to maintain arterial blood pressure. Despite inotropic support, 64% experienced at least one hypotensive episode. Concomitant inotropes decreased midazolam clearance by 33% • The relationship between plasma concentration of midazolam and blood pressure during hypothermia in patients without concomitant inotropic medication: MABP (mm Hg) = MABPbase (mm Hg)– 36.3 · Cp, MDZ (mg/l) • where MABPbase is the individual baseline MABP before the start of the midazolam infusion, and Cp, MDZ is the individual prediction of the midazolam plasma concentration in mg/l.
McAdams *et al. [30]*	Dexmedetomidine	94 blood samples	• Plasma concentrations rose gradually; near plateau levels (300 to 900 pg/mL) only after 12 to 24 h of infusion (>10 h after the infusion reached 0.4 μg/kg/h) • Upon discontinuation of dexmedetomidine infusion at 6h after rewarming, plasma concentration declined exponentially and remained detectable up to as long as 43 h after infusion stopped • PK parameters ⚬ CL (L/h/kg) 0.761 ± 0.155 ⚬ Vss (L/kg) 5.22±2.62 • Medication loss through sorption to the microbore tubing was observed over 18h of infusion. Average 5% cumulative loss of dose delivered over the 55–75 h infusion • Clearance was either comparable or lower, distribution volume was larger, and mean residence time or elimination half-life was longer in hypothermic neonates compared to normothermic neonates at similar gestational and postmenstrual ages
Favie *et al. [64]*	Morphine	853 blood samples	• Plasma concentrations varied for morphine (10–371 μg/L), for M3G (11–930 μg/L) and M6G (5–211 μg/L). • The parameter for morphine is estimated for 3.5 kg on PNA 0 days ⚬ Cl, l/h 0.899 (0.797–0.985) ⚬ Vd, l 8.88(7.87–9.92) ⚬ Interindividual variability Cl 47%, Vd 68% • GA and PNA were identified as covariates on morphine clearance but not on metabolite clearance ⚬ Morphine clearance was increased by 50% at PNA 5 days, compared to birth (an increase of 0.4%/h) ⚬ At birth, morphine clearance in a neonate with GA 36 weeks was 46% lower than GA 40 weeks, while clearance in a neonate with GA 42 weeks was 23% higher. • Morphine clearance during hypothermia was decreased by 21% (7%/°C) compared to normothermia. Metabolite clearance during hypothermia was reduced by 15% (5%/°C) • After rewarming, average morphine clearance was increased by 64% compared to clearance at the start of hypothermia • As clearance is not constant but increased over time, no steady state in morphine plasma concentration was reached in the first five days of life
Favie *et al*. [65]	Morphine, midazolam	192 patients	• Renal clearance (M3G, M6G): relative effect of PNA on clearance was 1.2%/hours of life • Hepatic intermediate-clearance group (morphine, midazolam, and OHM): relative effect of PNA on clearance was 0.5%/hours of life • Large interindividual variability: 72% for the high-clearance compounds and 55% for the intermediate-clearance compounds • The influence of temperature on clearance was only significant for the intermediate-clearance drugs • High correlation between clearance of M3G and M6G (96.2%) • Parameters ⚬ Morphine Cl, L/h 0.81(0.7–0.9), Temperature on Cl %/^o^C 7 (5.2–8.3) ⚬ Midazolam Cl, L/h 0.51(0.4–0.6), Temperature on Cl %/^o^C 7 (5.2–8.3)
Frymoyer *et al. [66]*	Morphine	160 blood samples	• Birthweight was highly predictive of CL_morphine_, CL_M3G_, and CL_M6G_ • Serum creatinine was a significant predictor of M3G and M6G clearance • At a morphine infusion of 10 μg/kg/h, only 54% of neonates receiving hypothermia achieved a concentration within the target range, and 46% had a morphine concentration >40 ng/ml. • M6G clearance was reduced by approximately 30%, while serum creatinine increases from 0.6 to 1.2 mg/ml for a 3.5 kg neonate. • Predicted morphine clearance for a 3.5 kg neonate receiving hypothermia was 0.8 L/h, which is almost 50% lower than that reported for a normothermic neonate of 1.4–1.5 L/h
Kali *et al. [68]*	Morphine		• There were no differences in the mean serum and CSF concentrations of morphine and its metabolites between the infants with and without liver dysfunction • There was no correlation between morphine serum concentrations and the two metabolites M3G and M6G, nor was there a correlation was between M3G and M6G • There was positive correlation between serum metabolite to morphine ratios: M6G/M and M3G/M (Spearman r = 0.9654; P < 0.0001).
Balduini *et al. [63]*	Melatonin		• Median basal serum melatonin concentrations of 21 pg/ mL • Highest plasma melatonin concentrations were obtained 3–12 hours after the end of the infusion • The measured peak concentration varied greatly among patients (0.08‐0.28 μg/mL) • Parameters ⚬ Individual mean Cmax was 0.27 ± 0.04 μg/ mL ⚬ T1/2 of 51 ± 36 hours (26 hours in the population analysis) ⚬ Vd of 5.7 ± 0.08 L ⚬ CL of 0.21 ± 0.07 L/h • Steady state can be reached after 4 infusions repeated every 24 hours
Roka *et al. [54]*	Morphine		• Morphine infusion rate and treatment with hypothermia strongly influenced serum morphine concentrations with little evidence of collinearity. • Median morphine CL: 0.69 mL/min/kg (normothermia) • Steady-state morphine CL (at 48 hours) was 0.89 mL/min/kg (normothermia) • Serum morphine concentrations reached a steady state after 24 hours in the normothermia infants, but they continued to increase in the hypothermia group

Abbreviations: Cmax, maximal serum concentration, CL, Clearance, Vd, Volume of distribution, T1/2, Elimination half‐life,M3G, morphine-3-glucuronide, M6G, morphine-6-glucuronide (M6G), GA, gestational age,PNA, postnatal age, Vss, steady state distribution volume

**Table 5:** Summary of studies which report cumulative doses of medication in neonates undergoing therapeutic hypothermia.

Author	Dose used	Sample Size	Results
Montaldo *et al. [125]*	Fentanyl Infusion 1–3μg/kg/h.	64	Cumulative dose of fentanyl within 72 hours 132 (120–144) μg/kg
Uner *et al. [135]*	Loading dose Morphine 100 μg/kg/h for 2h. Then infusion reduced to 20 μg/kg/h. If infant needed >50 μg/kg/h morphine infusion, midazolam was added at a dose of 20–50 μg/kg/h. Prior to procedures, fentanyl boluses 1–4 μg/kg was used.	17	• Cumulative dose of morphine during the 3 days of hypothermia was 2.09 ± 0.68 mg/kg • Fentanyl exposure from boluses for those neonates who had continuous morphine infusions: 18.3 ± 7.23μg/kg • Fentanyl exposure from continuous fentanyl infusions: 110.4 ± 17.08μg/kg.
Roka *et al. [54]*	Morphine Bolus dose 50–150 μg/kg followed by 5–30 μg/kg/h.	16	• Median cumulative morphine doses administered: 0.58–0.60 mg/kg/h • Serum morphine concentration was 292 ng/ml (24h -72h) in hypothermia group compared to 206ng/ml in the normothermia group despite no difference in cumulative dose or infusion rates • AUC for serum morphine concentrations over the entire study period was 18,608 ng/h per mL in the hypothermia group and 12,135 ng/h per mL in the normothermia group.
Mahdi *et al. [72]*	Intermittent morphine bolus 0.01–0.1 mg/kg while morphine infusion rates 0.01–0.04 mg/kg/h. Intermittent boluses of fentanyl 0.4–1.05 μg/kg, while infusion rates at 1 μg/kg/h.	153	• Cumulative doses of morphine received over the course of hypothermia ranged between 0–2 mg/kg • 48% of the total doses of opioids were administered within day 1, 36% within day 2, and 16% within day 3 • Cumulative dose of opioids correlated with lower skin temperature and lower Apgar scores at 10 min.
Lugli et al. [33]	Loading 2 μg/kg fentanyl bolus, followed by 0.5–2 μg/kg/h infusion rate	45	• The mean cumulative dose of fentanyl was 62 ± 18 μg/kg • Mean cumulative fentanyl dose was not significantly different among mild, moderate and severe encephalopathy • Fentanyl cumulative dose was not significantly different also between spontaneously breathing infants (59± 18μg/kg) and ventilated infants (66 ± 20μg/kg).
Gundersen *et al. [38]*	Not specified	282	• median cumulative opioid dose administered during the first week of life was 2121μg/kg
Welzing *et al. [37]*	Continuous infusion of midazolam at a dose of 30–100 μg/kg/h.	9	• Cumulative dose of midazolam 5.25 (3.2–6.7) mg/kg

#### Midazolam

There is high inter-individual variability of serum midazolam concentration in asphyxiated neonates during hypothermia [37]. However, pharmacokinetics of midazolam in neonates undergoing TH were similar to pharmacokinetics parameters in neonates under normothermic conditions [36, 37]. TH per se does not change the metabolic pathway of Midazolam, but metabolism may be severely altered in hepatic and renal impairment [37]. The typical half-lives of Midazolam varied between 5 hours without inotropes and 7.5 hours with inotropes due to a pharmacokinetic interaction between inotropes and Midazolam [36]. Due to concurrent hepatic and renal dysfunction, neonates with severe asphyxia may have decreased midazolam clearance contributing to systemic hypotension [36, 37].

#### Morphine

Hypothermia reduces the clearance of morphine and metabolites of morphine [65]. Based on simulation studies, Favie *et al*. reported that morphine loading of 50 μg/kg, followed by maintenance of 5 μg/kg/h is desirable to achieve a target plasma concentrations between 10–40 μg/L [65]. Nevertheless, there is considerable interpatient variability, along with the risk of dangerously high (>40 μg/l) serum concentrations in some patients [65]. While morphine has intermediate hepatic clearance, metabolites of morphine [M3G, morphine-3-glucuronide and M6G, morphine-6-glucuronide] have renal clearance [64, 69]. The clearance of morphine and its metabolites increases with postnatal age of the neonate [64]. A small study reported that continuous infusion of morphine at 25 μg/kg/h is tolerated well by hypothermic neonates and did not reach toxic serum concentration; however, morphine penetrates CSF at a higher concentration relative to glucuronide metabolites [68]. Population pharmacokinetic models indicate that hypothermia may affect the clearance of morphine and its glucuronide metabolites to a greater degree in neonates with lower weights than those with higher weights (estimated an exponent of 1.23) [66].

#### Clonidine

Intravenous Clonidine at a dose of 1 μg/kg every 8h administered over 30 mins during TH was reported as safe along with less opiate needs for shivering and agitation [67]. In contrary to population pharmacokinetic models derived from term neonates with a narcotic withdrawal syndrome, during TH the clearance of clonidine was reduced by 22% and volume of distribution was reduced by 28% [67]. Gauda *et al*. compared a historical cohort of hypothermic neonates who received morphine only, with neonates who received Clonidine every 8 hours during TH spent lesser time above the target temperature. Moreover, during the rewarming phase, neonates treated with Clonidine took 48% longer time to reach normal temperature (9h) versus controls (6h) [67].

#### Dexmedetomidine

Although not statistically significant, the mean clearance of Dexmedetomidine (0.91 ± 0.50 L/h/kg) for normothermic neonates without hypoxic-ischemic encephalopathy was higher than the mean clearance in hypothermic neonates (0.76 ± 0.16 L/h/kg) of similar gestational and postmenstrual age [30]. The authors suggest the need for a loading dose or initial rapid dose escalation to overcome the initial delay in achieving desired serum levels of Dexmedetomidine [30]. This is because, the predicted elimination half-life of Dexmedetomidine is about 7 hours in hypoxic-ischemic encephalopathy, compared to the elimination half-life of 3 hours for normothermic, non-encephalopathic neonates [30]. Therefore, Dexmedetomidine steady state is not achieved until about 28 hours (4 half-lives) after initiating or escalating the rate of infusion in neonates undergoing hypothermia as opposed to only 13 hours in normothermic neonates [30]. Loss of drug through adsorption to intravenous microbore tubing may further delay achieving a steady state concentration. It is believed to result in a 30% lower delivery of the medication than actual intended infusion rate during the first 6 hours of initiation [30].

#### Melatonin

One small study investigated the pharmacokinetics and safety of melatonin to use this drug as a neuroprotective agent [63]. The estimated elimination half‐life of melatonin is 51 ± 36 hours, estimated volume of distribution is 5.7 ± 0.08 L, and estimated clearance is 0.21 ± 0.07 L/hour [63]. The suggested dose of melatonin is 1‐5 mg/kg [63].

### Question 4. How do analgesia and sedation affect short- and long-term outcomes of neonates undergoing therapeutic hypothermia?

#### Short-term outcome (clinical)

There is an estimated decrease of 3.6 mmHg in mean arterial blood pressure with every 0.1 mg/l increase in serum midazolam concentration [36]. Inotropic support during the first three days was needed in 36% of unsedated neonates versus 46% of whom received opioids alone, 40% of whom received benzodiazepines alone, and 57% received opioids and benzodiazepines [48]. Of note, Dexmedetomidine, used as a sedative, compared to others, led to less hypotension and lower need for inotrope [70]. In general, neonates not exposed to sedation/analgesia had shorter durations of mechanical ventilation and hospital stay [62]. Hypothermic neonates who received opioids for 3–5 days or received a combination of opioid and benzodiazepine, had a longer median durations of ventilation [5 days versus 2 days] and hospital stay [12 days versus 11 days] compared to neonates who received either none or a shorter duration of opioids [1–2 days] [48, 60]. Almost 90% of infants exposed to opioids during TH were ventilated for the entire duration of therapy (median 95h), which strongly correlated with sedation duration [38]. In a comparative study, neonates in the dexmedetomidine group (n = 26) had similar efficacy in pain and agitation control compared to the fentanyl group (n = 19); however, the former was associated with decreased need for sedative bolus, shorter time to discontinuation of sedatives after rewarming, shorter time to extubation and resumption of feeds [31]. There was no difference in mortality and incidence of bradycardia, hypotension or apnea [31]. These findings were further confirmed by O’Mara *et al*. that Dexmedetomidine did not significantly impact heart rate, blood pressure, or cerebral saturations; rather, enteral feeding was initiated around 3 days, and full enteral feeds were attained by day of life 6 [26]. Although not associated with significant hemodynamic instability, Dexmedetomidine predominantly lowers heart rate nadir between 12-36h of life as compared to fentanyl monotherapy. For the neonates <35 weeks gestational age, the mean hourly heart rate nadir was slightly higher compared to neonates 36–38 weeks or >39 weeks [71].

#### Short-term outcome (neurological)

Both Dexmedetomidine and intermittent morphine use for sedation/analgesia during TH was associated with similar incidence of severely abnormal electroencephalogram (EEG) [11%] patterns, and extensive hypoxic-ischemic brain injury [11%] [29]. The cumulative dose of Fentanyl was not associated with normal, moderate or severe brain injury, even when corrected by the degree of encephalopathy [33]. When comparing a group of neonates receiving hypothermia with a group receiving hypothermia with morphine infusion, no difference was noted in the severity of brain injury [68]. In another small cohort study (N = 31), a higher dose of opioids was associated with lower odds of brain injury on MRI [beta coefficient -6.8, p = 0.01]. However, the odds ratio was pretty low, 0.001 (0–0.193) [72]. Neonates who receive a longer duration of opioids (3–5 days) tend to have slightly higher rates of severely abnormal EEG (21% versus 15%), Normal MRI (34% versus 31%), G tube feeds (4.3% versus 3.3%), need for supplemental oxygen at discharge (5.7% versus 0.9%) but lower rates of unadjusted mortality (12% versus 24%) compared to neonates who received shorter duration of opioids (1–2 days) [60]. After adjustment for severity of encephalopathy, opioid exposure of 3–5 days during TH remained independently associated with prolonged NICU stay and longer time on respiratory support and tube feedings at discharge [60]. In a comparative study, neonates in the dexmedetomidine group (n = 26) had lower incidence of seizures compared to the fentanyl group (n = 19); however, not statistically significant [31].

#### Long-term neurodevelopmental outcome

None of the studies were powered to study the association between sedation exposure and long-term outcome. In a comparative study of neonates receiving only hypothermia versus hypothermia with morphine, there was noted to be a reduction in death [5 (22.7%) versus 2 (8.7%), p = 0.24] or neurodevelopmental impairment at 18 months [17 (89.5%) versus 9 (72%), p = 0.28] in the TH plus morphine group compared to TH group, but was not statistically significant [68]. Natarajan *et al*. reported the rates of death/disability in infants with no exposure to sedation-analgesia (50%), a single agent at a one-time point (52%) and those with greater exposure (59%) [60]. There was no independent association between the sedation/analgesia exposure level and composite outcome [death or disability], adjusting for confounders [62]. A multicentre database analysis noted that neonates who undergo TH and are exposed to 3–5 days of opiate administration are more often referred to speech, occupational or physical therapy (18.1%) than neonates who received no opioids (10.5%) [60]. A subcohort (n = 186) of infants had outcomes data measured at 11 months of age, however, the duration of opioid exposure was had no association with death or neurodevelopmental impairment [60].

In a subgroup analysis of prospectively followed hypothermic neonates, those with the favourable long-term outcome (18–24 months) showed no difference in cumulative doses of morphine received on the first day of life [0.22 mg/kg] and first + second day of life [0.47 mg/kg] compared to neonates with unfavourable outcome, i.e. 0.19 mg/kg and 0.43 mg/kg respectively [73]. On the contrary, Meder *et al*. reported a significant difference in cumulative morphine doses at 84 hours between neonates with favourable outcomes [850 (760–990) μg] and abnormal outcomes [740 (380–740)μg] [74]. Gundersen *et al*. concluded that the cumulative dose of opioids administered during TH [median 2121 μg/kg] in the prospectively collected population cohort had no significant association with any of the domains of early childhood development [38].

## Discussion

### Pain and sedation assessment tools

Neonates are solely dependant on caregivers to interpret and manage pain and discomfort. Several standardized pain assessment tools have been applied to neonates undergoing TH, NPASS (Neonatal Pain, Agitation and Sedation Scale) being the most frequently used. Existing pain scales can be classified as (1) one-dimensional or behavioural, *e*.*g*. Échelle de Douleur et discomfort du Nouveauné (EDIN), Neonatal Facial Coding System (NFCS), Hartwig score, Visual Analog Scale (VAS), Neonatal Infant Pain Scale (NIPS), and (2) multidimensional, *e*.*g*. the NPASS and COMFORTneo scales, which incorporate physiological changes in addition to behavioural changes, making the assessment more comprehensive. None of the reported pain scales has been explicitly validated for encephalopathic neonates undergoing TH [75].

NPASS is a multidimensional instrument rating pain and sedation in 5 domains: crying, behaviour, facial expression, extremity tone, and vital signs [76]. NPASS has been validated for acute pain, prolonged pain and sedation at 23–30 weeks gestational age [77]. EDIN scale is a one-dimensional scale based on facial expression, movements, sleep, contact with nurses, and consolability [78]. Hartwig scale is validated for ventilated neonates and their response to suctioning and prolonged ventilation, such as grimacing, gross motor movements, and eye-opening [79]. COMFORT scale scores infants for level of alertness, degree of agitation, respiration, movement, muscle tone, facial expression, and vitals, showing adequate reliability with good construct validity for sedation but poor construct validity for pain [80]. The construct and target age group varies among different pain scales as follows: (i) EDIN (prolonged pain, 26–36 weeks and modified version 31–38 weeks), (ii) Hartwig (sedation in ventilated children 0–10 months), (iii) NFCS (Acute pain,1–12 months), shortened NFCS (Ventilated child, prolonged pain, 35 weeks to 18months), (iv) Observational VAS (Acute pain, 35 weeks to 4 years), (v) COMFORTneo (sedation, prolonged pain, 24–43 weeks) and NIPS (Acute pain, 27weeks to 7months) [75].

Commonly used neonatal pain scales are validated in specific neonatal subpopulations and have good psychomotor properties. The EDIN score is not validated for term neonates; NPASS and COMFORT have not been validated for ventilated neonates. Only the Hartwig, EDIN, and NFCS scales were validated for ventilated neonates. While the NFCS, NPASS, and COMFORT/COMFORT-B are validated for prolonged pain in term neonates, only NPASS and COMFORT/COMFORT-B are also validated for sedation [75]. The validity and reliability of scales vary between the scales and the different populations it was validated in, making an accurate assessment of pain and comparison between various centres impractical. Of all scales, NPASS, EDIN, COMFORT, and NFCS were rated as having the lowest risk of bias [75]. Due to encephalopathy and baseline neurological variance, there are practical challenges in assessing pain, especially by behavioural scales in hypothermic encephalopathic infants. In summary, two multidimensional scales (COMFORT/COMFORT-B and NPASS) appear to be the most well-suited monitoring tools for pain and sedation during hypothermia.

### Pharmacological and nonpharmacological pain management strategies

Sedation/analgesia is not universally used during TH since studies report that certain centres do not routinely administer medications to infants. Practice surveys report that 40–80% of all infants undergoing TH receive medication for sedation/analgesia. Upon secondary analysis of large hypothermia clinical trials, only 60% of neonates were exposed to sedation/analgesia; however, the use of sedation/analgesia and cumulative doses of drugs administered in the first three postnatal days progressively increased over years. For the studies that used sedation/analgesia, the most frequently used medications were Morphine, Fentanyl, Midazolam, and Dexmedetomidine, in descending order of frequency. Concomitant use of opiates and benzodiazepines is also prevalent. Two small comparative efficacy studies also suggested that Dexmedetomidine for sedation/analgesia leads to a decreased need for opioid bolus doses and a shorter time to extubation and resumption of feeds. The review further illustrates the wide variability in the dose ranges used for each medication and inconsistency in the choice of intermittent bolus and/or continuous infusions. Continuous infusion of Morphine, Fentanyl, and Midazolam varied between 8–60 μg/kg/hour, 1–5 μg/kg/hour and 50–400 μg/kg/hour, respectively. Studies that use Dexmedetomidine start with a slow infusion of 0.2–0.3 μg/kg/hour and titrate up to maximum doses of 1–2 μg/kg/hour.

Neonatal pain has been implicated in the development of excitotoxic brain damage and the disruption of normal brain development. TH’s lack of substantial benefit in reducing mortality in low-to-middle-income countries is attributed partly to the lack of optimum sedation/analgesia [81]. Moreover, preclinical studies have shown that even brief exposure to sedatives/analgesia in asphyxiated neonatal rats is associated with increased apoptosis of microglia, behavioural change, and mortality [82]. There appears to be a delicate balance between using sedation/analgesia to control associated pain and the neurotoxicity of the drugs by itself. Further research is needed to define optimal sedation/analgesia, compare the efficacy of different medications, and precisely titrate the doses according to the needs of individual infants. Nonpharmacological methods, such as cuddling and non-nutritive sucking, are gaining popularity because of their perceived lack of adverse effects and effectiveness in mitigating mild pain. Newer agents, such as Melatonin and Dexmedetomidine, potentially have additional neuroprotective effects and may be the preferred medication for sedation. Clinical data regarding their efficacy in neonates undergoing TH are limited [82, 83]. In summary, there is wide variability in practice and a paucity of well-designed studies comparing pharmacological pain control agents in neonates during hypothermia therapy. Future studies should focus on the effective use of nonpharmacological agents, either alone or in combination with other pharmacological agents, especially those with neuroprotective effects.

### Variability in pharmacokinetics

Few studies have measured the clearance of commonly used medications and highlighted the impact of hypothermia therapy, asphyxia-related hepatic and renal impairment, postnatal age, and birth weight on the clearance of medications. Morphine undergoes glucuronidation to M3G (no sedative properties) and M6G, which is pharmacologically active and a stronger sedative/analgesic than morphine [84]. The enzymatic activity of UDP glucuronosyl-transferase in neonates is one-tenth of its activity in adults but increases by 50% during the first few days after birth [84]. Both M3G and M6G undergo renal clearance. Therefore, hypothermia may reduce the clearance of both morphine as well as glucuronide metabolites, by decreasing hepatic and renal perfusion [66]. Morphine clearance increases during the first five postnatal days after birth, independent of the effect of temperature due to enzymatic maturation and recovery of organ [66]. Hence, lower doses will be need for sedation/analgesia in hypothermic neonates especially those with lower birth weight compared to term neonates without HIE. No pharmacokinetic studies of Fentanyl in hypothermic neonates have been reported.

Midazolam is another frequently used sedative in this population and its pharmacokinetic pattern have been widely studied in normothermic neonates. Midazolam is metabolised in the liver by cytochrome p450 to its hydroxy-metabolites [rate of 9:1] [37], which undergoes renal clearance [85]. The pharmacokinetics of Midazolam is not significantly affected by hypothermia per se. However, metabolism may be severely altered in asphyxia-induced hepatic and renal impairment [37]. The concomitant administration of inotropes prolongs the half-life of Midazolam due to a 33% decrease in clearance. There is high inter-individual variability of Midazolam concentrations in asphyxiated neonates with TH. Simultaneous hypoxic injury to the liver and kidneys in perinatal asphyxia, is likely to contribute to altered metabolism of Midazolam in HIE. It may have a profound impact because inadvertently high Midazolam concentrations has an increased risk of hypotension, heightened sedation, or potentially neurotoxicity on the developing brain. Neonatologists should be aware of the increased risk of adverse events during this period.

Central alpha-2 adrenergic receptor agonist (Clonidine and Dexmedetomidine) modify the central thermoregulatory setpoints for shivering and have been reported effective in postoperative shivering [86]. Additionally, α2-adrenergic agonists may provide mild analgesia, sedation without respiratory depression, and potentially neuroprotective to immature brain as demonstrated by pre-clinical models of brain injury [87]. They also have well-known opioid-sparing effects. In the context of TH, α2-adrenergic agonists stabilize core body temperature in the optimal therapeutic range by suppressing counter-regulatory defence mechanisms such as shivering and non-shivering thermogenesis [88, 89]. By inducing thermal tolerance, α2-adrenergic agonists may decrease stress and agitation during therapy. Recent studies have focussed on the pharmacokinetics of Dexmedetomidine, a lipophilic drug which undergoes glucuronidation and hydroxylation in the liver and excreted by the kidneys. Asphyxiated newborns undergoing hypothermia had higher volume of distribution than non-asphyxiated, normothermic newborns. Hence, a loading dose or initial rapid dose escalation is needed for Dexmedetomidine to achieve effective plasma levels owing to a prolonged elimination half-life and minor adsorptive losses in the tubing. Hence, clinicians would have to aware of the slower onset of action, when starting with a Dexmedetomidine infusion.

### Clinical and neurological outcome

Sedative/analgesic medications are commonly associated with bradycardia, hypotension, need for invasive ventilation, delayed establishment of full enteral feeds, and prolonged length of NICU stay. When compared with opiates, few studies report Dexmedetomidine’s safety profile, its opioid-sparing effect, and bradycardia. Short-term adverse effects such as hypotension, prolonged need for respiratory support, and delayed feeding were the most profound in neonates who received a combination of opioids and benzodiazepines. However, none of these studies were designed to study the direct relationship between exposure to sedation/analgesia and short-term adverse outcome. Therefore, a causal relationship cannot be deduced. Considering the baseline risk of brain injury due to hypoxia-ischemic insult, no independent association was noted between the severity of brain injury on neuroimaging and cumulative opioid exposure. Although not statistically significant, Dexmedetomidine was associated with a lower incidence of seizures. There is dearth of studies adequately powered to investigate the long-term neurological outcomes of this population’s exposure to sedation/analgesia. In addition, the study designs were heterogeneous, and the findings were contradictory.

### Strengths and limitations

The included studies were largely cross-sectional and observational, primarily designed to investigate the safety and efficacy of the sedation/analgesic medications in this population. There is no randomized control trial that studied the efficacy of pain scales or individual pharmacokinetic agents in sedation/analgesia management. Most studies had small sample sizes and heterogeneous study designs; therefore, the findings have limited generalizability. Inconsistencies in how pain and distress were objectively assessed in encephalopathic neonates further contribute to the discrepancies in the study findings. The review was limited to English language articles due to the practical challenges of systematically finding and evaluating relevant non-English publications and gray literature, acknowledging the size of this review.

## Conclusion

Considerable variability in administering sedation/analgesia during induced hypothermia in neonates across different centres calls for standardized practice recommendations. Despite the widespread use of TH, it is not routine practice to administer sedation/analgesia or to use standardized pain assessment tools. Without tools validated in this population, COMFORT and NPASS are the most suitable tools for assessing sedation and prolonged pain. Opioids and benzodiazepines were the most frequently used medications. Dexmedetomidine has recently gained particular attention in this population, given its better safety profile, less respiratory depression, and potential additive effects, including opioid sparing, stability of core body temperature, shivering control and neuroprotection. The significant inter-individual variability in drug levels of the same drug administered to different neonates at the same dose due to variable impacts of body temperature, end-organ dysfunction, postnatal age, body weight on drug metabolism/clearance calls for more precise control of drug dosing. Future prospective studies will need to study the independent effect of sedation/analgesia on long-term outcomes, adjusting for the impact of the underlying severity of brain injury.

## Supporting information

S1 Appendix PRISMAScR checklist. (PDF)

S2 Appendix Search strategy. (DOCX)

S3 Appendix Data extraction tool. (DOCX)
