# Supplementary material for: Analgesia and sedation strategies in neonates undergoing whole-body therapeutic hypothermia: A scoping review
Source: PLoS One. 2023 Dec 7;18(12):e0291170. doi: 10.1371/journal.pone.0291170 (PMC10703341; doi:10.1371/journal.pone.0291170)
Supplement: S2 Appendix — (DOCX) [file pone.0291170.s002.docx]

**Appendix II: Search Strategy**

*Embase (Ovid)*

| Date of Search January 05,2023 | | |
| --- | --- | --- |
| Search | Query | Records retrieved |
| 1 | analgesia/ or analgesi*.mp. or analgesic agent/ | 358126 |
| 2 | sedation/ or sedat*.mp. | 147108 |
| 3 | pain/ or nociceptive pain/ | 351009 |
| 4 | pain control.mp. | 25472 |
| 5 | discomfort.mp. | 97279 |
| 6 | stress.mp. or physiological stress/ | 1524884 |
| 7 | cold pain.mp. or cold/ | 25912 |
| 8 | 1 or 2 or 3 or 4 or 5 or 6 or 7 | 2343940 |
| 9 | benzodiazepines.mp. or benzodiazepine derivative/ | 61758 |
| 10 | opioid.mp. or opiate/ | 182760 |
| 11 | barbiturate.mp. or barbituric acid derivative/ | 23152 |
| 12 | dexmedetomidine/ | 16661 |
| 13 | midazolam/ | 56582 |
| 14 | morphine equivalent dose/ or morphine/ or morphine derivative/ or morphine-induced antinociception/ or morphine sulfate/ | 119177 |
| 15 | fentanyl citrate/ or fentanyl/ or fentanyl derivative/ | 75258 |
| 16 | lorazepam/ | 29640 |
| 17 | paracetamol/ or paracetamol sulfate/ or paracetamol derivative/ | 107036 |
| 18 | hydromorphone/ | 12054 |
| 19 | alfentanil/ | 7054 |
| 20 | methadone/ | 35793 |
| 21 | meperidine.mp. | 3455 |
| 22 | chloral hydrate/ | 6969 |
| 23 | clonidine/ | 43518 |
| 24 | 9 or 10 or 11 or 12 or 13 or 14 or 15 or 16 or 17 or 18 or 19 or 20 or 21 or 22 or 23 | 557505 |
| 25 | pain management.mp. | 48047 |
| 26 | cold stress/ | 20804 |
| 27 | (pain adj2 (manag* or treat* or therap*)).mp. | 99709 |
| 28 | 8 or 25 or 26 or 27 | 2368428 |
| 29 | infant/ | 670663 |
| 30 | newborn care/ or newborn/ or newborn intensive care/ | 602577 |
| 31 | neonat*.mp. | 411101 |
| 32 | 29 or 30 or 31 | 1244937 |
| 33 | induced hypothermia/ or hypothermia/ or experimental hypothermia/ or profound induced hypothermia/ | 50735 |
| 34 | therapeutic hypothermia.mp. | 7634 |
| 35 | cooling.mp. | 53721 |
| 36 | (hypothermi* adj2 (induce* or therap* or mild or treat*)).mp. [mp=title, abstract, heading word, drug trade name, original title, device manufacturer, drug manufacturer, device trade name, keyword heading word, floating subheading word, candidate term word] | 23768 |
| 37 | 33 or 34 or 35 or 36 | 100113 |
| 38 | brain hypoxia/ | 11175 |
| 39 | newborn hypoxia/ or brain ischemia/ or hypoxic ischemic encephalopathy/ | 170001 |
| 40 | perinatal asphyxia/ | 5975 |
| 41 | ((anoxia or ischem* or hypoxic*) adj2 (brain or cerebral or encephalopathy*)).mp. [mp=title, abstract, heading word, drug trade name, original title, device manufacturer, drug manufacturer, device trade name, keyword heading word, floating subheading word, candidate term word] | 190091 |
| 42 | 38 or 39 or 40 or 41 | 204713 |
| 43 | 37 or 42 | 297663 |
| 44 | 24 or 28 | 2709353 |
| 45 | 32 and 43 and 44 | 3166 |
| 46 | limit 45 to (human and english language) | 1954 |
| 47 | limit 46 to yr="2005 -Current" | 1676 |

*Ovid Medline(R) 1996 to November 29,2022*

| Date of Search January 05,2023 | |  |
| --- | --- | --- |
| Search | Query | Records retrieved |
| 1 | analgesia/ or analgesi*.mp. or analgesic agent/ | 221480 |
| 2 | sedation/ or sedat*.mp. | 86951 |
| 3 | pain/ or nociceptive pain/ or pain control.mp. or discomfort.mp. or stress.mp. or physiological stress/ or cold pain.mp. or cold/ or pain management.mp. or cold stress/ or (pain adj2 (manag* or treat* or therap*)).mp. | 1411701 |
| 4 | benzodiazepines.mp. or benzodiazepine derivative/ or opioid.mp. or opiate/ or barbiturate.mp. or barbituric acid derivative/ or dexmedetomidine/ or midazolam/ or morphine equivalent dose/ or morphine/ or morphine derivative/ or morphine-induced antinociception/ or morphine sulfate/ or fentanyl citrate/ or fentanyl/ or fentanyl derivative/ or lorazepam/ or paracetamol/ or paracetamol sulfate/ or paracetamol derivative/ or hydromorphone/ or alfentanil/ or methadone/ or meperidine.mp. or chloral hydrate/ or clonidine/ | 255703 |
| 5 | infant/ or newborn care/ or newborn/ or newborn intensive care/ or neonat*.mp. | 1370154 |
| 6 | induced hypothermia/ or hypothermia/ or experimental hypothermia/ or profound induced hypothermia/ or therapeutic hypothermia.mp. or cooling.mp. or (hypothermi* adj2 (induce* or therap* or mild or treat*)).mp. [mp=title, book title, abstract, original title, name of substance word, subject heading word, floating sub-heading word, keyword heading word, organism supplementary concept word, protocol supplementary concept word, rare disease supplementary concept word, unique identifier, synonyms] | 79698 |
| 7 | brain hypoxia/ or newborn hypoxia/ or brain ischemia/ or hypoxic ischemic encephalopathy/ or perinatal asphyxia/ or ((anoxia or ischem* or hypoxic*) adj2 (brain or cerebral or encephalopathy*)).mp. [mp=title, book title, abstract, original title, name of substance word, subject heading word, floating sub-heading word, keyword heading word, organism supplementary concept word, protocol supplementary concept word, rare disease supplementary concept word, unique identifier, synonyms] | 100724 |
| 8 | 1 or 2 or 3 or 4 | 1761376 |
| 9 | 6 or 7 | 176334 |
| 10 | 5 and 8 and 9 | 1185 |
| 11 | limit 10 to (english language and humans) | 650 |
| 12 | limit 46 to yr="2005 -Current" | 433 |

*Cochrane Library*

| ID | Date of Search January 05,2023 | Hits |
| --- | --- | --- |
| Search | Query | Records retreived |
| #1 | MeSH descriptor: [Analgesia] explode all trees | 8651 |
| #2 | analgesi* OR sedati* with Publication Year from 2005 to 2022, in Trials | 70025 |
| #3 | Conscious Sedation | 2697 |
| #4 | Hypnotics | 4754 |
| #5 | Narcotics | 2427 |
| #6 | Analgesics, Opioid | 11005 |
| #7 | narcotic* or opioid* or barbital or "chloral hydrate" or dexmedetomidine or diazepam or lorazepam or midazolam or pentobarbital or phenobarbital or alfentanil or fentanyl or morphine or hydromorphone or meperidine or methadone | 71912 |
| #8 | Pain Management | 39322 |
| #9 | pain NEAR/2 (manag* or treat* or therap*) | 40411 |
| #10 | cold stress | 1073 |
| #11 | Infant, Newborn | 23062 |
| #12 | newborn OR neonat* OR infant* | 87878 |
| #13 | Hypothermia, Induced | 1888 |
| #14 | hypothermi* NEAR/2 (induce* or therap* or mild or treat*) | 2245 |
| #15 | targeted temperature management | 263 |
| #16 | Hypoxia-Ischemia, Brain | 337 |
| #17 | (anoxia OR ischemi* OR hypoxi*) NEAR/2 (brain OR cerebral OR encephalopath*) | 8877 |
| #18 | {OR #1-#10} | 153008 |
| #19 | {OR #11-#12} | 87878 |
| #20 | {OR #13-#15} | 2601 |
| #21 | {OR #16-#17} | 8887 |
| #22 | {AND #18-#21} | 37 |

*CIHAHL (EBSCO)*

| Date of Search January 05,2023 | | |
| --- | --- | --- |
| Search | Query | Records retrieved |
| S1 | (MH "Pain Management") OR (MH "Acute Pain Control (Saba CCC)") OR (MH "Pain Control (Saba CCC)") OR (MH "Nociceptive Pain") | 13,148 |
| S2 | (MH "Hypnotics and Sedatives") OR (MH "Sedatives, Barbiturate") OR (MH "Conscious Sedation") OR (MH "Sedatives, Nonbarbiturate") OR (MH "Sedation") | 14,355 |
| S3 | (MH "Hypothermia, Induced") OR (MH "Hypothermia Treatment (Iowa NIC)") OR (MH "Hypothermia (Saba CCC)") OR (MH "Hypothermia (NANDA)") OR (MH "Hypothermia") | 8,555 |
| S4 | (MH "Infant, Newborn") OR (MH "Infant, High Risk") OR (MH "Intensive Care Units, Neonatal") OR (MH "Intensive Care, Neonatal") | 151,306 |
| S5 | S1 OR S2 | 27,346 |
| S7 | (MH "Hypoxia-Ischemia, Brain") OR (MH "Hypoxia-Ischemia, Brain, Neonatal") OR (MH "Cerebral Ischemia") OR (MH "Hypoxia, Brain") OR (MH "Cerebral Ischemia, Transient") | 21,159 |
| S8 | S3 OR S7 | 28,737 |
| S9 | S4 AND S5 AND S8 | 17 |

*Web of Science*

| Date of search | Date of Search January 05,2023 |
| --- | --- |
| Search string | **analgesi* OR sedat* OR narcotic* OR opioid* OR barbital OR "Chloral hydrate" OR dexmedetomidine OR diazepam OR lorazepam or midazolam OR pentobarbital OR phenobarbital OR alfentanil OR fentanyl OR morphine OR hydromorphone OR meperidine OR methadone OR "pain manag*"** (All Fields) or **pain NEAR/3 (manag* or treat* or therap*)** (Title) or **pain NEAR/3 (manag* or treat* or therap*)** (Abstract) AND **infant* or newborn or neonat*** (All Fields) AND  **hypothermi* OR "targeted temperature management"** (All Fields) or **hypothermia* NEAR/3 (induce* OR therap* OR treat*)** (Title) or **hypothermia* NEAR/3 (induce* OR therap* OR treat*)** (Abstract) AND **"hypoxia ischemia"** (All Fields) or **(anoxia OR ischemia OR hypoxi*) NEAR/3 (brain* or cerebral or encephalopath*)** (Title) or **(anoxia OR ischemia OR hypoxi*) NEAR/3 (brain* or cerebral or encephalopath*)** (Abstract) |
| Number of results | 905 |

*SCOPUS*

| Date of search | Date of Search January 05,2023 |
| --- | --- |
| Search string | TITLE-ABS-KEY ( analgesi* OR sedat* OR narcotic* OR opioid* OR barbital OR "Chloral hydrate" OR dexmedetomidine OR diazepam OR lorazepam OR midazolam OR pentobarbital OR phenobarbital OR alfentanil OR fentanyl OR morphine OR hydromorphone OR meperidine OR methadone OR "pain manag*" ) AND TITLE-ABS-KEY ( infant* OR newborn OR neonat* ) AND TITLE-ABS-KEY ( hypothermi* OR "targeted temperature management" OR "cooling" ) AND TITLE-ABS-KEY ( "hypoxia ischemia" OR "encephalopathy" OR "perinatal asphyxia" ) |
| Number of results | 395 |
