# Supplementary material for: Analgesia and sedation strategies in neonates undergoing whole-body therapeutic hypothermia: A scoping review
Source: PLoS One. 2023 Dec 7;18(12):e0291170. doi: 10.1371/journal.pone.0291170 (PMC10703341; doi:10.1371/journal.pone.0291170)
Supplement: S3 Appendix — (DOCX) [file pone.0291170.s003.docx]

| **Article Information** | |
| --- | --- |
| First author last name |  |
| Title |  |
| Journal name |  |
| Year of publication |  |
| **Characteristics of Included Studies** | |
| Main aim of the study |  |
| Start date |  |
| End date |  |
| Study design |  |
| Geographical location |  |
| Sedative agent used |  |
| Route of administration |  |
| Dose used |  |
| **Type of Delivery** |  |
| Pain and stress assessment tool used |  |
| Serum levels of medication measured (Yes or No) |  |
| Neurodevelopmental outcome assessed (Yes or No) |  |
| Participants | |
| Total number of participants in the cooling arm |  |
| Population description |  |
| **Results Extraction** | |
| Effect of hypothermia on the pharmacokinetics of the medication |  |
| Dosage range used for the medication |  |
| Non-pharmacological comfort measures used |  |
| Main finding of the article |  |
|  |  |

Appendix III: Data Extraction Tool
